# Unearthing the Unknown Causes of Meningitis

**DOI:** 10.4269/ajtmh.20-0502

**Published:** 2020-06-22

**Authors:** Senjuti Saha

**Affiliations:** 1 Child Health Research Foundation, Dhaka, Bangladesh;; 2 Department of International Health, Johns Hopkins Bloomberg School of Public Health, Baltimore, Maryland

Scientists around the world are painstakingly developing a road map to defeat meningitis by 2030.^1^ Once called “phrensy” or “brain fever,” meningitis can be caused by a multitude of pathogens. The three main culprits, and the primary focus of WHO surveillance platforms, are *Haemophilus influenzae* type b (Hib), *Streptococcus pneumoniae*, and *Neisseria meningitidis*. Before the advent of antibiotics, meningitis by these pathogens killed almost all children they affected; this changed drastically with timely use of appropriate antibiotics starting in the mid-1900s. Next, with the global rollout of the Hib vaccine beginning in the 1990s, rates of Hib meningitis have significantly reduced in most countries.^2^ Pneumococcal and meningococcal vaccines are also being introduced in many populations, and obvious positive impacts are emerging. However, as these vaccines are taken up by populations and meningitis surveillance systems are strengthened with incorporation of new technologies, we are uncovering large gaps in the current meningitis literature, specifically related to incomplete microbiological diagnosis of suspected meningitis cases.

In an article published in this issue of the *Journal*, Kwambana-Adams^3^ and team suggest roles for several additional bacterial and viral pathogens in causing meningitis in < 5-year-old children. Using 711 cerebrospinal fluid (CSF) specimens collected from five different countries across West Africa, Senegal, Ghana, Togo, Nigeria and Niger, this study showed that a large proportion of meningitis cases remained microbiologically undiagnosed after molecular testing (triplex PCR) for the three pathogens noted previously. To extend surveillance for many additional potential pathogens, the team used a customized TaqMan Array Card (TAC) to simultaneously detect 15 bacteria, 17 viruses, one fungus, and two protozoans using quantitative polymerase chain reaction (qPCR). With this approach, microbiological diagnosis of probable meningitis causes was increased by 18% compared with triplex PCR (from 2% to 20%).

Among the 19 different pathogens detected, *Escherichia coli*, *Plasmodium* spp, *S. pneumoniae*, *Staphylococcus aureus*, and *Klebsiella pneumoniae* were the most common, followed by several viruses including parvovirus B19 and dengue. All detected pathogens have previously been associated with neurological infections, but are not commonly screened using molecular techniques, highlighting the importance of this study. The epidemiological profiles of some of these pathogens were different than anticipated; for example, Gram-negative pathogens like *E. coli* and *K. pneumoniae* are usually associated with neonatal meningitis, but the authors detected them in older children. In addition, in about 4% of the specimens, more than one pathogen was detected in the CSF, a phenomenon not commonly reported in the literature. A proportion of these “uncommon” findings may be explained by underlying conditions like severe malnutrition found in the study populations. It is also noteworthy that most of the previous epidemiological data on meningitis come from developed nations, where demographic and clinical profiles of cases are markedly different.

Although very important and timely, the findings of this study should be considered within the context of several limitations. This was a retrospective etiology detection study, not specifically designed to collect and preserve specimens for sensitive molecular testing by the TAC technology. Lack of stringent collection and preservation methods may have hampered the quality of the specimens, as DNA and RNA are susceptible to degradation, and as contamination may have been introduced.^4^ The 711 CSF samples were gathered from five countries with different demographic profiles of cases, making comparison between countries and generalization of the results difficult, if not impossible. Finally, and perhaps most importantly, there were no control (healthy/true negative) specimens included in the study. A large study that used TACs to test more than 10,000 blood and respiratory samples to determine causes of neonatal sepsis depicted the importance of including healthy control specimens and rigorous statistical modeling to distinguish between true pathogens and false positives.^5^ This caveat is also evident in this study; DNA of pathogenic bacteria and parasites were detected and considered an etiology in many cases with very low white blood cell count (< 10/cmm), whereas specimens from cases of bacterial meningitis usually have higher white blood cell count.^6^ With techniques as sensitive as the qPCR, the risk of false positives is high and should be considered with utmost diligence to avoid misinterpretations. A recent study from Bangladesh that sequenced total nucleic acid present in about 100 CSF samples of children with and without meningitis illustrated the abundance of “pathogenic” bacterial nucleic acid on the skin of the children, in hospital and laboratory environments, and in testing reagents, further illustrating the importance of incorporating control specimens to negate false positives.^7^ These limitations should be critically considered when designing future studies.

Despite these limitations, this study demonstrates key gaps in our knowledge on etiology of meningitis and proposes a molecular technique that can be incorporated into ongoing and future surveillance studies. With increasing use of over-the-counter antibiotics, culture-positivity rates are declining, and, hence, molecular techniques to detect bacterial etiologies are becoming increasingly important. Furthermore, most surveillance studies in resource-poor settings do not screen for viruses. A technique that can probe for many different pathogens simultaneously may play a vital role in comprehensive attribution of etiologies of meningitis. However, the panel of pathogens to be screened for in such a test will likely differ between countries/populations, as circulating pathogens vary by geography, and the number of pathogens that can be tested using one TAC assay will be limited by cost and available sample volume. Hence, such multiplex qPCRs will need to be customized based on data on circulating pathogens in specific populations. For instance, in this study, despite screening for 35 different pathogens, 80% of cases remained microbiologically undiagnosed, illustrating the need for supplementary methods to detect an even more diverse range of pathogens. Recent reports suggest that techniques like unbiased next-generation RNA metagenomic sequencing, albeit resource- and cost extensive and not scalable in the immediate future in resource-poor settings, may be able to guide the design of custom TACs and increase the rate of detection.^7^

The global estimates of meningitis stand at 10.6 million cases and 288,000 deaths every year, with the vast majority occurring in low- and middle-income countries.^8,9^ In addition, about a quarter of bacterial meningitis cases that survive live with lifelong neurological disability.^10^ Increasing numbers of reports are revealing that up to 85% of WHO-defined meningitis cases evade etiology detection despite using all standard laboratory methods, in line with Kwambana-Adams findings.^3,11,12^ As a global community, if we indeed want to defeat meningitis by 2030, now is the time to invest and conduct studies on understanding all causes of meningitis; so, we can implement comprehensive and evidence-based treatment protocols and prevention strategies.
